# Interfacial Enhancement of Polyethylene Fiber-Reinforced ECC via Multi-Walled Carbon Nanotubes Functionalization

**DOI:** 10.3390/nano16120714

**Published:** 2026-06-10

**Authors:** Baolin Peng, Chonggen Pan, Yuxin Huang, Huiqing Wang, Jian Geng, Yedong Chen, Xiangkun Meng, Youpeng Duan

**Affiliations:** 1 College of Civil Engineering and Architecture, Zhejiang University, No. 866 Yuhangtang Road, Xihu, Hangzhou 311400, China; 22312228@zju.edu.cn; 2 School of Civil Engineering, NingboTech University, No. 1 Qianhu South Road, Yinzhou, Ningbo 315100, China; 15960542263@163.com (H.W.); gengjian@whut.edu.cn (J.G.); 18867626850@163.com (Y.C.); 3 College of Civil Engineering, Qingdao University of Technology, Qingdao 266033, China; hyx15689113367@163.com (Y.H.); m15552921052@163.com (X.M.); duanyoupeng2002@163.com (Y.D.)

**Keywords:** polyethylene fibers, MWCNTs, surface modification, ECC, mechanical properties

## Abstract

Polyethylene (PE) fibers are promising reinforcements for engineered cementitious composites (ECC); however, their highly hydrophobic nature and inherent chemical inertness limit their reinforcing effectiveness. This study investigated the use of different types of multi-walled carbon nanotubes (MWCNTs) to modify PE fibers under varying immersion times. Microstructural characterizations were conducted to investigate the effects of MWCNTs type and immersion time on the surface properties of PE fibers, while mechanical testing was undertaken to evaluate the mechanical performance of the resulting fiber-reinforced cementitious composites. MWCNTs were found to form a uniform coating on the surface of the reinforced PE fibers, resulting in a reduction in water contact angle from 164.2° to 118.4° and an increase in oxygen contents by 242.27%. With increasing immersion time, the single-fiber pull-out strength improved by up to 40.48%, with an optimal modification duration of 8 h. The MWCNTs modified PE fibers were found to increase the 28-day uniaxial tensile strength and three-point bending strength of the cementitious composites by up to 16.17% and 6.96%, respectively, while exhibiting negligible effects on compressive strength. This study implies that MWCNTs can effectively enhance surface wettability and mitigate surface inertness of PE fibers, thereby enhancing the overall performance of ECC.

## 1. Introduction

Engineered cementitious composites (ECC) are advanced civil engineering materials characterized by high ductility, multiple cracking behavior, and excellent durability, and have been widely applied in seismic-resistant structures, crack control, and long-life infrastructure [1]. Polyethylene (PE) fibers, as a key reinforcing component, possess ultra-high tensile strength, high elastic modulus, and superior corrosion resistance, demonstrating great potential for application in ECC [2,3,4]. However, the intrinsic low surface energy, strong chemical inertness, and superhydrophobic nature of pristine PE fibers result in poor compatibility with the inorganic cementitious matrix, inevitably leading to the formation of a loose and weak interfacial transition zone (ITZ) at the fiber–matrix interface, which becomes the weakest link in the composite system [5]. Under external loading, PE fibers are prone to premature debonding and pull-out from the matrix, preventing full utilization of their high strength and stiffness, and thereby limiting the enhancement of ECC ductility and load-bearing capacity [6,7,8].

Surface modification of PE fibers has therefore become a central research focus. Existing modification approaches include plasma treatment, chemical oxidation, nanomaterial coating, and polymer grafting. Liu et al. [9] effectively improved the strain-hardening behavior of ECC by coating fiber surfaces with a silane coupling agent. Belgacemi et al. [10] introduced polar functional groups via potassium dichromate oxidation, significantly enhancing interfacial bonding. Li et al. [11] employed chromic acid to oxidize PE fiber surfaces, successfully overcoming their surface inert-ness. Another study on natural rubber-based composites similarly demonstrated that appropriate surface modification can markedly improve the mechanical load-transfer efficiency between PE fibers and the matrix [12]. Wang [13] enhanced the interfacial performance between PE fibers and rubber composites using an ozone-assisted UV grafting method. While these methods can strengthen interfacial bonding to varying degrees, they generally suffer from complicated processing, relatively high costs, and, more critically, the risk of introducing structural defects that compromise the intrinsic tensile strength of the fibers. The chemical oxidation method, for instance, involves corrosive reagents that may etch and degrade the fiber surface; plasma treatment requires specialized equipment, and its effects may decay over time; and polymer grafting often involves multi-step syntheses that are difficult to scale. Therefore, rational surface functionalization of PE fibers to strengthen interfacial bonding with the cementitious matrix and optimize stress transfer is of great theoretical significance and practical value for advancing their application in high-performance cementitious composites [14, 15].

Multi-walled carbon nanotubes (MWCNTs) offer an attractive alternative, owing to their unique spatial architecture, extremely high specific surface area, exceptional mechanical properties, and good thermal stability [16]. Their distinctive tubular nanostructure can form nanoscale physical anchoring sites on polymer fiber surfaces, while functionalized variants—such as hydroxylated and carboxylated MWCNTs—provide active sites for chemical bonding with cement hydration products, thereby enhancing interfacial bonding strength [17]. Alrekabi et al. [18] incorporated MWCNTs in combination with steel fibers, achieving simultaneous improvements in flexural strength, tensile strength, and toughness. Raki et al. [19] reported that carbon nanotubes can increase the Vickers hardness of cement paste by approximately sixfold, the Young’s modulus by 2.3-fold, and flexural performance accordingly. In addition, both PE fibers and carbon nanotubes exhibit strong hydrophobicity, which provides an intrinsic driving force for interfacial self-assembly in aqueous environments [20]. Leveraging this property, aqueous self-assembly enables the uniform and stable coating of carbon nanotubes on PE fiber surfaces without the need for chemical crosslinking agents or destructive treatments. Compared with the above-mentioned methods, the MWCNTs-based aqueous self-assembly approach is mild and environmentally benign, avoids fiber damage, and is relatively simple to implement—making it a promising route for engineering applications [21,22]. For these reasons, MWCNTs are selected as the modifier in this study.

Current studies on PE fiber surface modification mainly focus on the aforementioned methods; however, the application of carbon nanotubes to modify PE fibers for cementitious composites remains limited, particularly with respect to the quantitative relationships among nanotube type, immersion duration, fiber surface characteristics, and macroscopic mechanical performance [23,24,25]. This study, therefore, aims to investigate the effect of MWCNTs on the microstructure of PE fibers and the influence of MWCNTs-modified PE fibers on the mechanical performance of ECC by: (1) modifying PE fibers using three types of MWCNTs and characterizing their surface morphology, wettability, chemical composition, and interfacial bonding properties; and (2) assessing the effects of MWCNTs-modified PE fibers on the compressive, tensile, and flexural performance of ECC, and proposing an optimized modification strategy for engineering applications.

## 2. Experimental Materials and Methods

### 2.1. Raw Materials

**Figure 1:**
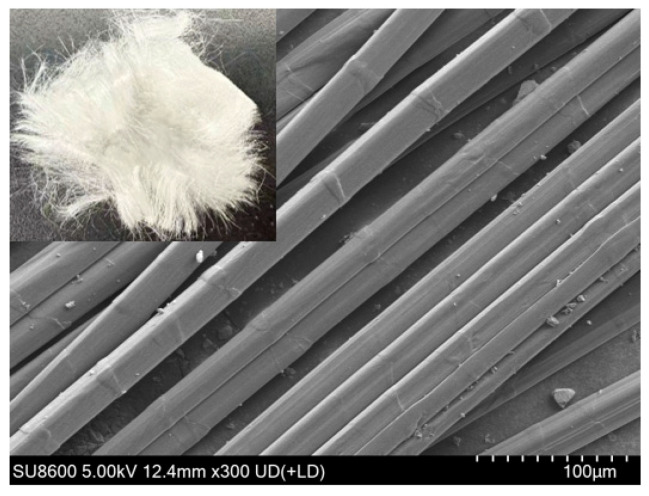
Surface and microstructure of PE fibers.

The raw materials used in this study included: P.O 42.5 ordinary Portland cement (Conch brand) with a residue on an 80 μm sieve not exceeding 10%; Class II fly ash (Class F) produced by Beilun Power Plant, having a fineness of 8.6% residue on a 45 μm sieve; natural fine sand supplied by Fengping Mineral Products Co., Ltd. (Yantai, China), with a fineness modulus of 1.5 and a maximum particle size of 0.6 mm; and a polycarboxylate superplasticizer manufactured by Ningbo Zhongshuike Chemical Technology Co., Ltd. (Ningbo, China), with a water-reduction rate of 25%. The reinforcing fibers used were polyethylene (PE) fibers produced by Beijing Quantum Tiandi New Materials Technology Co., Ltd. (Beijing, China), the macroscopic and microscopic morphologies of which are shown in Figure 1, and the properties are presented in Table 1.

**Figure 2:**
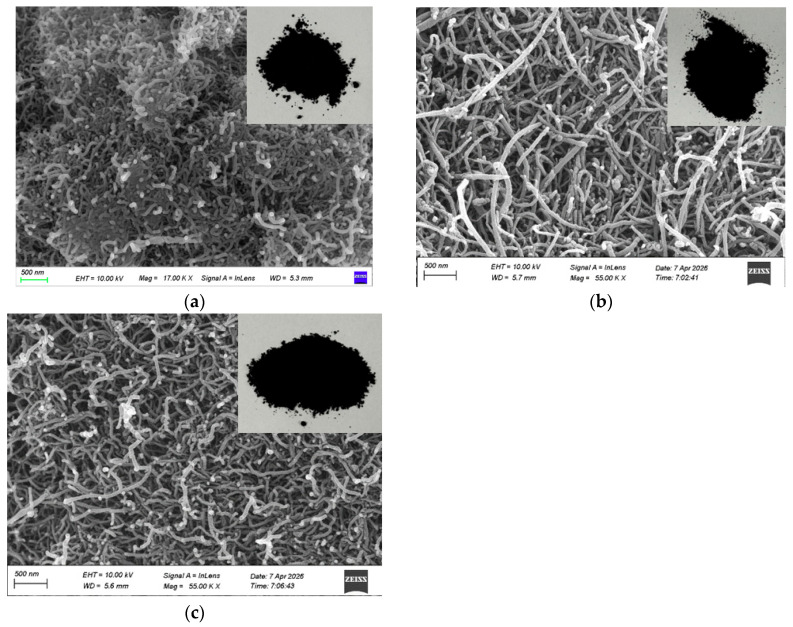
Appearance and SEM images of (**a**) MWCNTs-A; (**b**) MWCNTs-OH; (**c**) MWCNTs-COOH.

The MWCNTs used in this study were supplied by Suzhou Tanfeng Graphene Technology Co., Ltd. (Suzhou, China), including pristine MWCNTs (MWCNTs-A), hydroxylated MWCNTs (MWCNTs-OH), and carboxylated MWCNTs (MWCNTs-COOH). Their macroscopic appearance and microscopic morphology are shown in Figure 2, and their detailed properties are listed in Table 2.

### 2.2. Fiber Modification Mechanism

PE fibers are typical superhydrophobic polymer materials with extremely low surface energy. MWCNTs also possess highly hydrophobic graphene-based structures. Based on their shared hydrophobic characteristics, this study adopts an aqueous self-assembly strategy to achieve uniform coating of MWCNTs on the PE fiber surface, with the underlying mechanism governed by hydrophobically driven interfacial adsorption [12].

**Figure 3:**
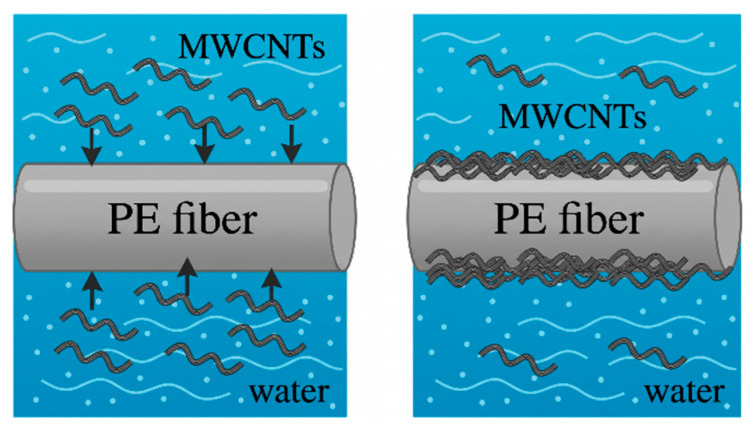
Schematic diagram of the principle of MWCNTs modified PE fiber.

When MWCNTs are uniformly dispersed in an aqueous medium, water molecules form highly ordered cluster-like structures upon contact with hydrophobic surfaces, leading to a reduction in system entropy. To minimize the water–hydrophobic interfacial area and achieve thermodynamic stability, hydrophobic components tend to spontaneously aggregate [24]. During this process, the PE fiber surface acts as a macroscopic hydrophobic substrate, while MWCNTs serve as nanoscale hydrophobic particles, resulting in physical adsorption driven by hydrophobic interactions. As a polar medium, water effectively repels hydrophobic particles, promoting the spontaneous and uniform deposition of MWCNTs onto the PE fiber surface, thereby forming a stable nanoscale coating. This process is illustrated in Figure 3. The approach does not require chemical crosslinking agents or destructive treatment of the fibers, representing a green and efficient functionalization method based on intrinsic material properties.

### 2.3. Fiber Modification Process

Based on interfacial adsorption theory, the modification of PE fibers was carried out according to the following procedure:

(1) MWCNTs suspensions (MWCNTs-A, MWCNTs-OH, and MWCNTs-COOH) were prepared separately at a concentration of 4 kg/m^3^. Based on preliminary experiments, this concentration was selected because it offers a relatively high MWCNT content, good dispersion (i.e., low agglomeration), and favorable economic efficiency;
(2) The PE fibers were cleaned using an ultrasonic cleaner for 10 min to remove surface impurities;
(3) The cleaned PE fibers were immersed in the suspensions to ensure complete submersion, followed by mechanical stirring at a speed of 100 r/min. The stirring was conducted intermittently, with 10 min of stirring followed by 10 min of in cycles, to prevent fiber agglomeration caused by prolonged continuous stirring;
(4) After immersion for 2 h, 4 h, 8 h, and 24 h, respectively, the PE fibers were removed and drained on a sieve;
(5) The fibers were then dried in an oven at 80 °C for 24 h and subsequently collected for further use.

### 2.4. Sample Preparation

The mix proportions of the cementitious matrix used in this study are presented in Table 3. For the preparation of fiber-reinforced cementitious composites, the fiber content was fixed at 1.5% by volume of the cementitious matrix. The mixing procedure was as follows: the dry powders were first mixed with 30% of the total fibers for 2 min, followed by the slow addition of 80% of the total water and the superplasticizer, and mixed for 5 min. After achieving a uniform mixture, the remaining 70% of the fibers and the remaining 20% of the water were added and mixed for an additional 3 min. The specimens were cured in a standard curing room. Three replicate specimens were prepared for each group.

The unmodified PE fibers were designated as the PE group, while the PE fibers modified with MWCNTs-A, MWCNTs-OH, and MWCNTs-COOH were denoted as the M-PE-A, M-PE-OH, and M-PE-COOH groups, respectively. The immersion durations were set at 2 h, 4 h, 8 h, and 24 h. Detailed group information is provided in Table 4.

### 2.5. Testing Methods

(1) Uniaxial Compressive Test

According to GB/T 17671-2021 [26], cubic specimens with dimensions of 40 mm × 40 mm × 40 mm were prepared for compressive testing. A STYE-300E automatic cement flexural and compressive testing machine (Zhejiang Tugong Instrument Manufacturing Co., Ltd., Shaoxing, Zhejiang, China) was used, with continuous and uniform loading applied at a rate of 2.4 kN/s.

(2) Three-Point Bending Test

Specimens with dimensions of 40 mm × 40 mm × 160 mm were prepared. The tests were conducted using a WA-50 electronic universal testing machine (Jinan Yongke Testing Instrument Co., Ltd., Jinan, Shandong, China), with a span length of 100 mm and a loading rate of 0.5 mm/min.

(3) Uniaxial Tensile Test

The specimens had dimensions of 330 mm × 60 mm × 30 mm. A WA-50 electronic universal testing machine was used, and deformation was measured using a YYU-80-25-SH displacement extensometer (Chengdu Ruiceta Testing Technology Co., Ltd., Chengdu, Sichuan, China). The extensometer was first attached to the central region of the dog-bone specimen using hooks, and the upper and lower grips of the testing machine were aligned to ensure coplanarity.

Loading was applied under displacement control at a rate of 0.5 mm/min. Tensile stress was calculated as the applied load divided by the cross-sectional area of the gauge section, while tensile strain was determined as the displacement over the gauge length. Stress–strain curves were obtained from the calculated stress and strain data during the tensile loading process.

(4) Single-Fiber Pull-Out Test

**Figure 4:**
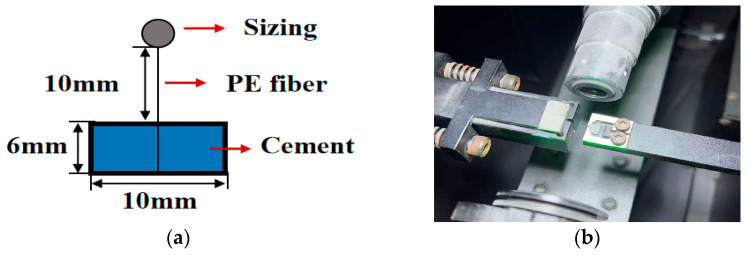
(**a**) Principal and (**b**) experimental setup of single-filament pull-out test.

A single PE fiber with a length of 50 mm was vertically embedded into a small cementitious matrix block with a thickness of 6 mm. After curing for 28 days, one end of the fiber was bonded with hot-melt adhesive, as shown in Figure 4. The test was conducted using an FSF003 high-precision fiber mechanical testing system (Shanghai Aifeisi Precision Instrument Co., Ltd., Shanghai, China) under displacement control, with a loading rate of 2 μm/s. The load and displacement data during the pull-out process were recorded simultaneously.

(5) Scanning Electron Microscopy (SEM) and Energy Dispersive Spectroscopy (EDS)

Microstructural morphology and elemental composition were analyzed using a Guoyi Quantum 4000Pro (China), a ZEISS GeminiSEM 360 (Germany), and an Oxford EBSD c swift system. Specimens were taken from fractured samples after the three-point bending test. Samples with dimensions of 10 mm × 10 mm × 5 mm were cut using a cutting machine, and the fracture surfaces were preserved for analysis.

(6) Raman Spectroscopy Analysis

Raman spectroscopy was employed to characterize the evolution of surface functional groups on PE fibers before and after modification. The principle of using Raman spectroscopy to evaluate interactions between heterogeneous materials is based on the fact that, when subjected to external stress or interfacial constraints, structural distortion occurs within the material, leading to shifts in characteristic Raman peaks associated with molecular vibrational modes. These shifts can be used to infer changes in the strength of chemical bonding or physical adsorption at the interface. A HORIBA LabRAM HR Evolution Raman spectrometer (Japan) was used to analyze the interactions between MWCNTs attached to the surface of modified PE fibers and the PE fibers themselves.

(7) Contact Angle Measurement

The contact angle of the fibers was measured using a JY-PH3 contact angle goniometer (China). Deionized water was used as the test liquid. Using the sessile drop method, three different locations were selected on each sample surface, and three droplets of deionized water with a volume of 0.6 μL were deposited at each location. The average contact angle was then calculated.

## 3. Results and Discussion

### 3.1. Monofilament Drawing Performance

**Figure 5:**
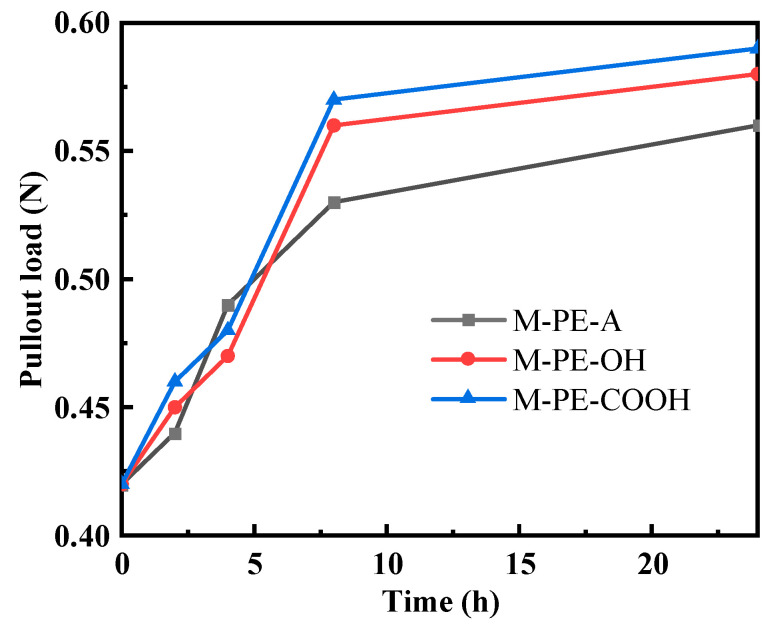
Maximum pull-out load of MWCNTs modified PE fiber monofilaments.

Single-filament pull-out tests were conducted on both modified and unmodified PE fibers embedded in a cementitious matrix to evaluate the effect of MWCNT modification on the interfacial bonding strength. The results are presented in Table 5, and the maximum pull-out loads are shown in Figure 5.

At a fiber immersion time of 2 h, the single-fiber pull-out strength of the M-PE-A, M-PE-OH, and M-PE-COOH groups increased by 4.76%, 7.14%, and 9.52%, respectively, compared with the PE group. At 4 h, the corresponding increases were 16.67%, 11.90%, and 14.29%, respectively. When the immersion time reached 8 h, the pull-out strength increased by 26.19%, 33.33%, and 35.71%, respectively. At 24 h, the increases further rose to 33.33%, 38.10%, and 40.48%, respectively. These results indicate that the enhancement in single-fiber pull-out strength becomes more pronounced with increasing immersion time, reflecting stronger interfacial bonding between the fibers and the cementitious matrix [27]. All types of MWCNTs-modified PE fibers improve the pull-out strength within the matrix, with the M-PE-COOH group exhibiting higher values than the M-PE-A and M-PE-OH groups.

### 3.2. Uniaxial Compressive Strength

**Figure 6:**
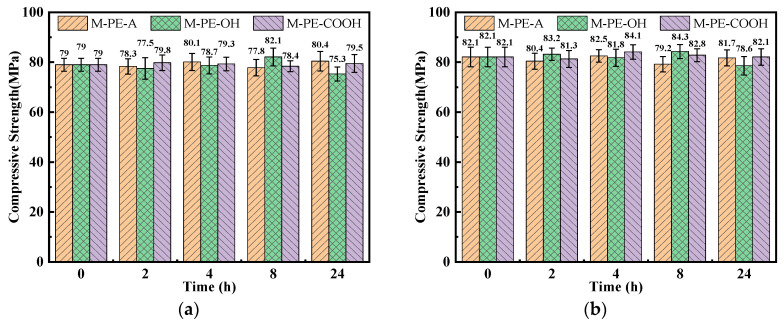
Compressive strength at (**a**) 7 d; (**b**) 28 d.

The uniaxial compressive strength results of cement-based composites reinforced with PE fibers modified by three types of MWCNTs at different immersion times are presented in Figure 6. The results indicate that MWCNTs-modified PE fibers have no significant effect on compressive strength, and the influence of fiber immersion time is likewise negligible. Moreover, only a marginal increase in compressive strength is observed from 7 d to 28 d. This can be attributed to the incorporation of fly ash in the cementitious matrix. Owing to its low pozzolanic reactivity, the secondary hydration of fly ash contributes only limited strength gain during the 7–28 d curing period; most fly ash particles remain unreacted and function primarily as physical fillers. Meanwhile, cement hydration is largely completed within 7 days, and the subsequent hydration rate slows significantly. These combined factors result in a limited strength in-crease from 7 d to 28 d. Regarding the role of fibers under compressive loading, the stresses in cement-based materials are primarily carried by the rigid cementitious skeleton. As flexible reinforcements, fibers mainly provide auxiliary confinement. Com-pressive failure is governed by splitting induced by transverse tensile strain; although fibers can restrain lateral deformation to some extent, this effect is limited relative to the direct action of compressive stress. Furthermore, fibers introduce an interfacial transition zone (ITZ) within the matrix. Modification of PE fibers enhances fiber–matrix bonding, thereby improving stress transfer and enabling the reinforcing effect of the fibers. However, the dispersion uniformity of fibers in the matrix directly affects the distribution of interfacial defects. The reduced hydrophobicity of modified PE fiber increases their tendency to agglomerate during mixing, leading to poor dispersion, which may adversely affect compressive strength. Considering the combined effects of these factors, no significant change in compressive strength is observed.

### 3.3. Uniaxial Tensile Properties

**Figure 7:**
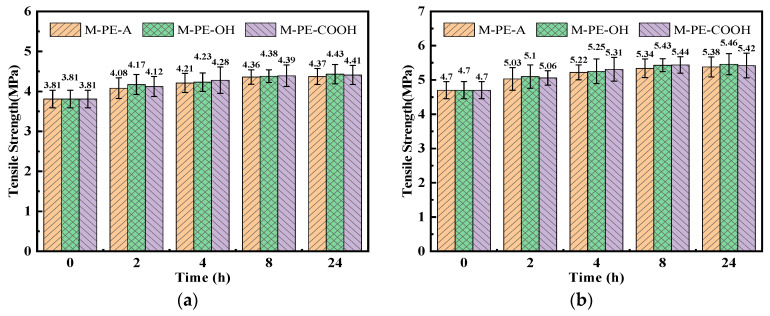
Uniaxial tensile strength at (**a**) 7 d; (**b**) 28 d.

**Figure 8:**
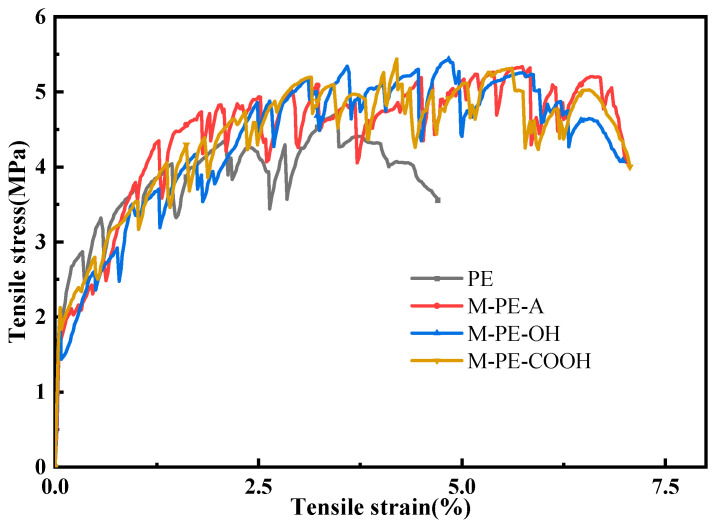
Uniaxial tensile stress–strain curves of MWCNTs-modified PE fiber specimens.

The uniaxial tensile strength test results of cement-based composites reinforced with PE fibers modified by three types of MWCNTs at different immersion times are presented in Table 6 and Figure 7. For the 28-day specimens, when the fiber immersion time was 2 h, the tensile strength of the M-PE-A, M-PE-OH, and M-PE-COOH groups increased by 7.02%, 8.51%, and 7.66%, respectively, compared with the PE group, while the tensile strain increased by 25.74%, 21.70%, and 27.02%, respectively. At an immersion time of 4 h, the tensile strength increased by 11.06%, 11.70%, and 12.98%, respectively, and the tensile strain increased by 44.04%, 41.06%, and 40.00%, respectively. When the immersion time reached 8 h, the tensile strength increased by 13.62%, 15.53%, and 15.74%, respectively, while the tensile strain increased by 49.36%, 48.51%, and 50.43%, respectively. At 24 h, the tensile strength increased by 14.47%, 16.17%, and 15.32%, respectively, and the tensile strain increased by 50.64%, 49.57%, and 51.49%, respectively. These results indicate that PE fibers modified with different MWCNTs enhance both tensile strength and tensile strain. Moreover, the improvement becomes more pronounced with increasing fiber immersion time and stabilizes after 8 h. Figure 8 presents the tensile stress–strain curves of specimens with fibers modified for 8 h and cured for 28 d. Compared with the unmodified PE fiber specimens, the modified PE fiber specimens exhibited a higher number of cracks with smaller crack widths and a more uniform crack distribution, indicating a significantly improved deformation capacity. Additionally, a significant increase in tensile strength was observed from 7 d to 28 d, which can be attributed to the progressive enhancement of fiber–matrix bonding with curing age. Among the three types of MWCNTs, the M-PE-OH and M-PE-COOH groups exhibit slightly higher tensile strength than the M-PE-A group, indicating that MWCNTs-OH and MWCNTs-COOH are more effective than MWCNTs-A in enhancing tensile performance [28].

### 3.4. Three-Point Bending Performance

**Figure 9:**
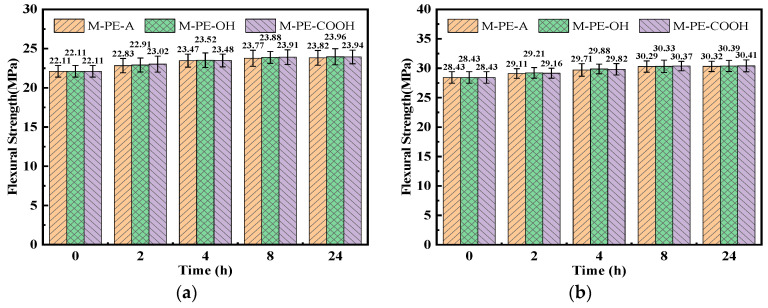
Three-point bending strength at (**a**) 7 d; (**b**) 28 d.

**Figure 10:**
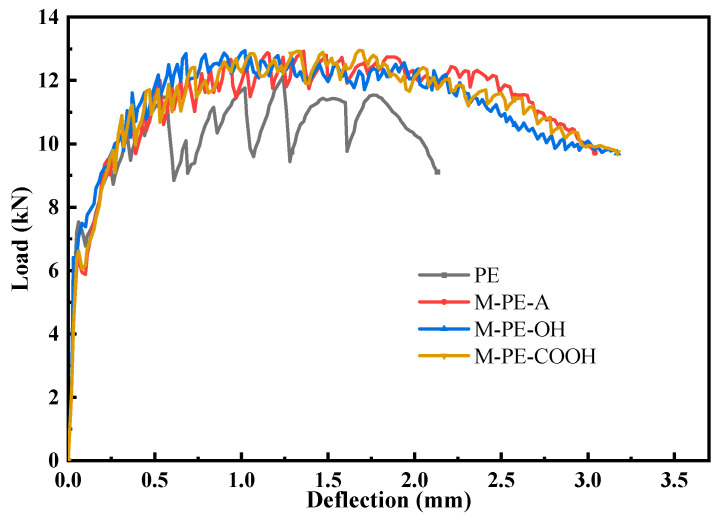
Three-point bending load–deflection curves of MWCNTs-modified PE fiber specimens.

The three-point bending test results of cement-based composites reinforced with PE fibers modified by three types of MWCNTs at different immersion times are presented in Table 7 and Figure 9. For 28-day specimens, when the fiber immersion time was 2 h, the three-point bending strength of the M-PE-A, M-PE-OH, and M-PE-COOH groups increased by 2.39%, 2.74%, and 2.57%, respectively, compared with the PE group, while the deflection increased by 21.60%, 23.94%, and 25.82%, respectively. At an immersion time of 4 h, the bending strength increased by 4.50%, 5.10%, and 4.89%, respectively, and the deflection increased by 30.99%, 36.62%, and 33.80%, respectively. When the immersion time reached 8 h, the bending strength increased by 6.54%, 6.68%, and 6.82%, respectively, while the deflection increased by 42.72%, 48.83%, and 49.77%, respectively. At 24 h, the bending strength increased by 6.65%, 6.89%, and 6.96%, respectively, and the deflection increased by 46.01%, 51.64%, and 49.30%, respectively. These results indicate that PE fibers modified with different MWCNTs improve both the three-point bending strength and deflection, with the M-PE-OH and M-PE-COOH groups exhibiting slightly higher bending strength than the M-PE-A group. Moreover, the enhancement becomes more pronounced with increasing fiber immersion time and stabilizes after 8 h. Figure 10 presents the three-point bending load–deflection curves of specimens with fibers modified for 8 h and cured for 28 d. The modified PE fiber specimens developed multiple fine cracks with reduced crack width and more uniform crack distribution, demonstrating a markedly improved deformation capacity. In addition, a clear growth in bending strength was observed from 7 d to 28 d, which can be attributed to the evolution of the fiber–matrix interface during curing, i.e., the progressive strengthening of the interfacial transition zone between fibers and the surrounding matrix as hydration advances.

### 3.5. Fiber Surface Micromorphology

**Figure 11:**
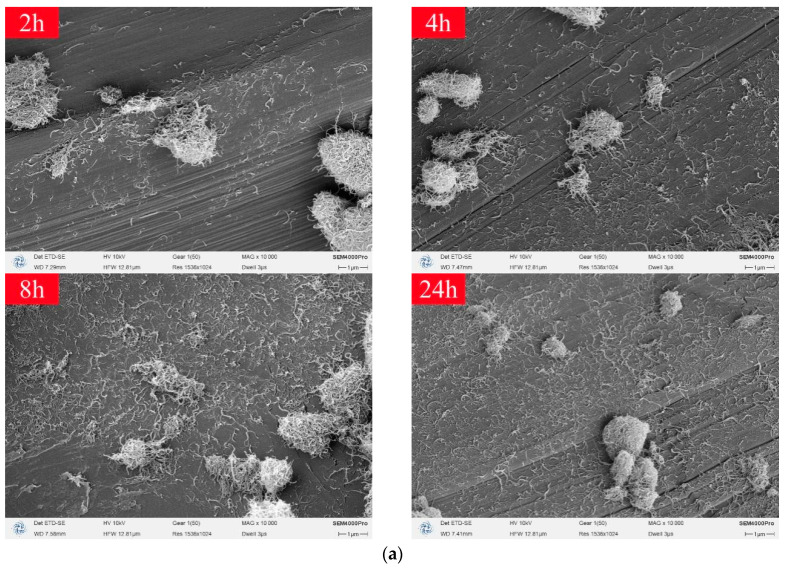

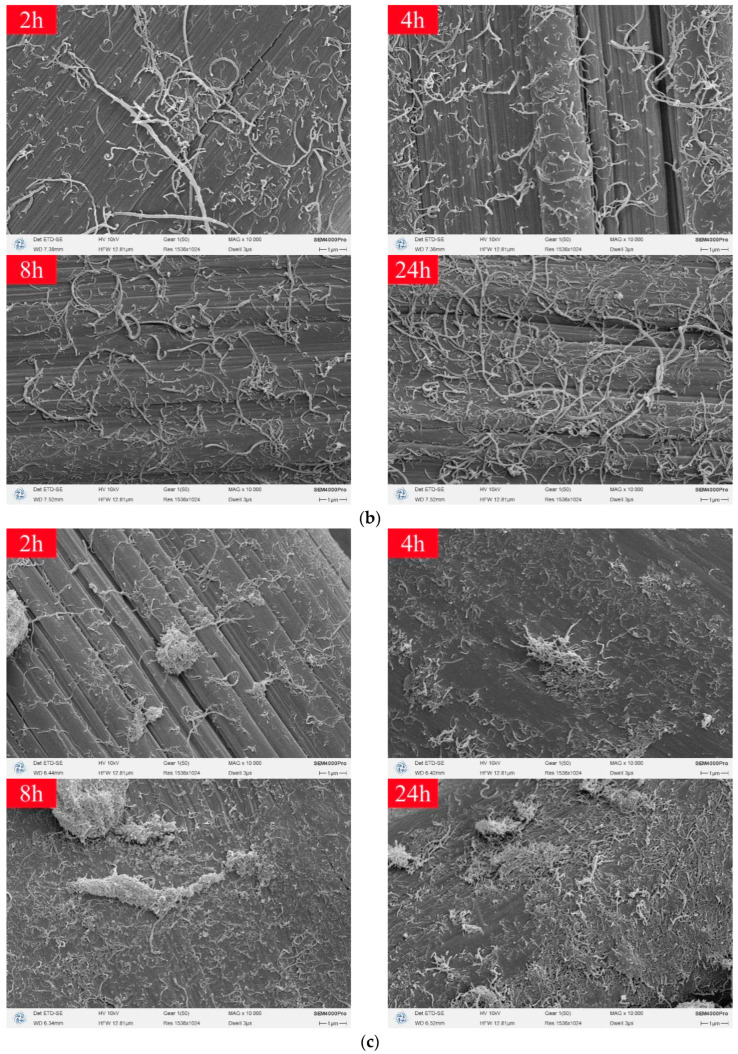
SEM image of (**a**) MWCNTs-A-modified PE fiber; (**b**) MWCNTs-OH-modified PE fiber; (**c**) MWCNTs-COOH-modified PE fiber.

The surface morphology of different MWCNTs-modified PE fibers treated under varying immersion times is presented in Figure 11. All types of MWCNTs were observed to form a three-dimensional coating on the surface of PE fibers. This coating can improve the surface properties of PE fibers by increasing their surface roughness and thus enhancing physical friction during the pull-out process [29]. The coating layer significantly increases the contact area and physical interactions between the fibers and the matrix, thereby strengthening the fiber–matrix interface. With increasing immersion time, all three types of MWCNTs exhibited a gradual increase in surface coverage on the PE fibers along with an increasingly uniform distribution. Compared with the MWCNTs-A and MWCNTs-COOH groups, in which a few agglomerates were present, the MWCNTs-OH group achieved a thinner coating layer on the PE fiber surface without any apparent agglomeration, suggesting that MWCNTs-OH exhibits the best dispersion performance among the three types of MWCNTs. This superior dispersion can be explained by the fact that hydroxyl groups form a dense and stable hydrogen-bond network with water molecules, building a robust hydration layer around each individual CNT that prevents inter-tube contact through steric repulsion. Meanwhile, hydroxyl groups preferentially interact with water rather than forming in-ter-tube crosslinks. In contrast, pristine MWCNTs (MWCNTs-A) are highly hydrophobic and thus agglomerate strongly in water. MWCNTs-COOH, although capable of generating electrostatic repulsion through ionization, are susceptible to charge screening by ions in the solution; moreover, carboxyl groups can readily form hydro-gen-bond bridges between adjacent tubes, which in turn promotes agglomeration.

### 3.6. Fiber Surface Wetting Properties

The contact angle is defined as the angle between the tangent to the gas–liquid interface and the solid–liquid interface at the three-phase contact line, and it is an important geometric parameter for quantitatively evaluating the wettability of solid surfaces. In general, a smaller contact angle indicates a stronger tendency of the liquid to spread on the solid surface and thus better wettability. Conversely, a larger contact angle corresponds to poorer wettability. In this study, a contact angle goniometer was employed to measure the static contact angles of distilled water on both unmodified PE fibers and MWCNTs-modified PE fibers, in order to systematically investigate the effects of MWCNTs type and immersion duration on the wettability behavior of the modified fiber surfaces.

**Figure 12:**
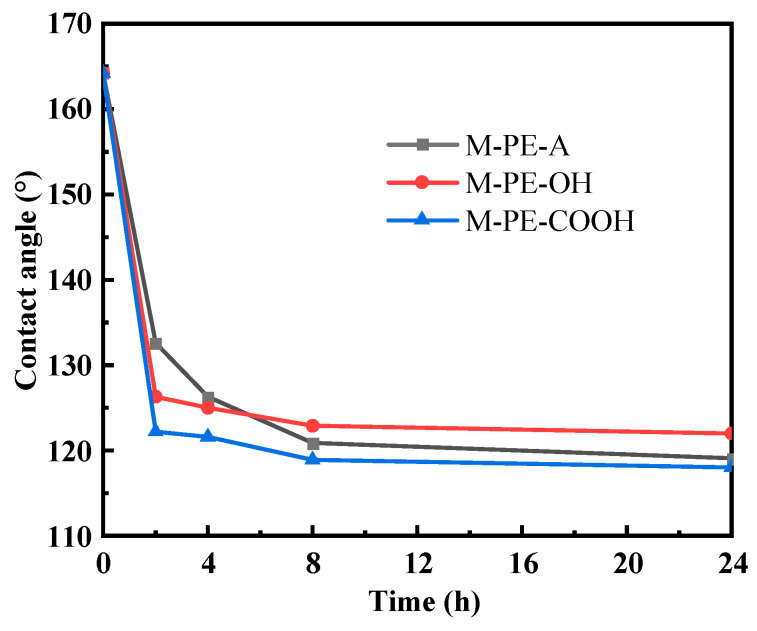
Contact angle variation of MWCNTs-modified PE fibers.

The contact angle test results of MWCNTs-modified PE fibers are presented in Table 8, and the variation trends are shown in Figure 12. The contact angle was found to decrease significantly in the initial 2 h immersion from 164.2° to a range of 122.2–132.6°, followed by a gradual decrease to a near stable ranges of 118.9–122.9° after 8 h, of which the M-PE-COOH group exhibits the most pronounced reduction.

These results indicate that MWCNTs can effectively enhances the surface wettability of PE fibers, and the modification approaches completion after 8 h. The improved wettability facilitates the spreading and diffusion of water or alkaline pore solutions on the fiber surface, thereby promoting the deposition and growth of cement hydration products. This enables the fiber surface to act as a heterogeneous nucleation site for hydration reactions, ultimately strengthening the interfacial interaction between the fibers and cement hydration products [30].

**Figure 13:**
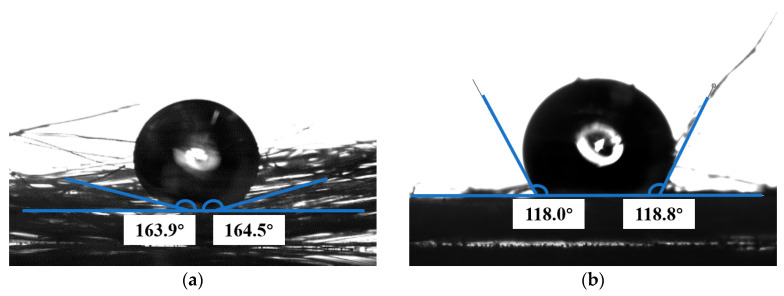
Contact angle of (**a**) PE fiber; (**b**) M-PE fiber.

A mixed MWCNTs suspension with a ratio of MWCNTs-A:MWCNTs-OH:MWCNTs-COOH = 6:1:3 and an immersion time of 8 h was therefore tested to modify PE fibers, optimizing cost and performance. In the initial stage of immersion, the surface of the PE fiber has a large number of exposed adsorption sites, allowing for MWCNTs to attach rapidly, so the amount of attached MWCNTs increases significantly with time. As the nanotubes gradually cover the fiber surface, the available sites decrease, and the already attached nanotubes create steric hindrance and repulsive effects, making further adsorption increasingly difficult; meanwhile, the desorption rate also rises as the amount of attached MWCNTs grows. After about 8 h, adsorption and desorption reach a dynamic equilibrium, and the fiber surface is essentially fully occupied and tends toward saturation, so the attachment amount no longer increases with time. In addition, if the nanotubes in the suspension undergo slow agglomeration over time, the number of effective adsorption units decreases, which can also cause the system to enter this plateau stage even earlier. The resulting MWCNTs-PE fibers exhibited a contact angle of 118.4°, as shown in Figure 13. In the subsequent study, all modified PE fibers were prepared using this method and are denoted as M-PE fibers.

### 3.7. Chemical Composition of Fiber Surface

**Figure 14:**
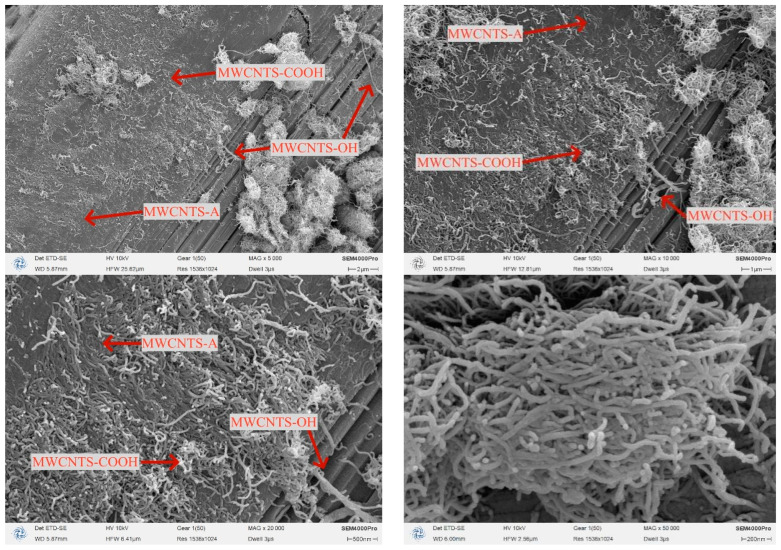
SEM images of M-PE fibers at different fiber ratios.

**Figure 15:**
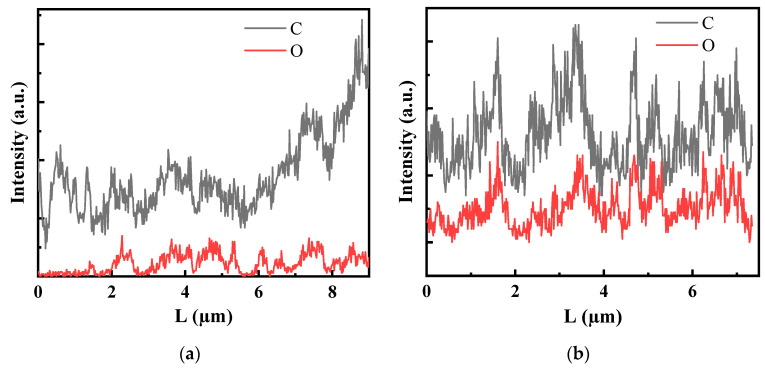
C and O content on the surface of (**a**) PE fiber; (**b**) M-PE fiber.

The SEM images of M-PE fibers are shown in Figure 14. SEM showed that the modified PE fibers are coated with a layer of material. Elemental compositions on the surfaces of PE fibers and MWCNTs-modified PE fibers were analyzed using EDS line scanning, and the results are presented in Table 9. The results showed that modification with MWCNTs increased the surface oxygen content in the order of MWCNTs-OH (390.72%), mixed MWCNTs (242.27%) and MWCNTs-OH (55.33%), compared with unmodified PE fibers with 2.91 mass %. No change to the oxygen content with MWCNTs-A modified PE fibers [31]. Figure 15 presents the elemental compositions of PE fibers and M-PE fibers.

**Figure 16:**
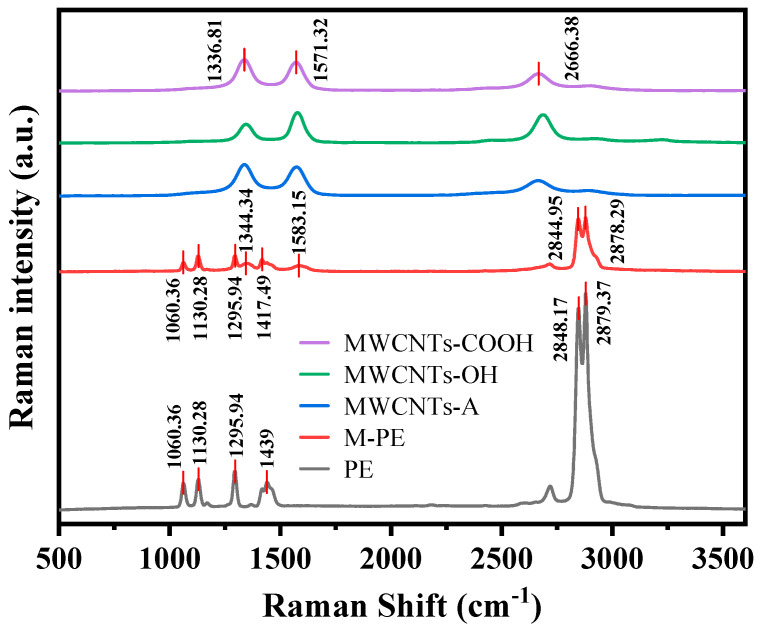
Raman spectroscopy results.

Figure 16 presents Raman spectra of MWCNTs, MWCNTs-OH, MWCNTs-COOH, unmodified PE fibers, and M-PE fibers. The unmodified PE fibers exhibit characteristic Raman peaks at 1060, 1130, 1296, 1439, 2848, and 2879 cm^−1^, corresponding to C-C backbone stretching vibrations and the twisting, bending, and stretching vibrations of -CH_2_- groups. After modification with MWCNTs, two new characteristic peaks appear at 1344 cm^−1^ and 1583 cm^−1^ in the Raman spectra of M-PE fibers. These peaks correspond to the D-band and G-band vibrational modes of carbon atoms in MWCNTs, indicating that MWCNTs have been successfully attached to the PE fiber surface. Compared with the characteristic peaks of free MWCNTs at 1337 cm^−1^ and 1571 cm^−1^, the D and G bands of MWCNTs shift toward higher wavenumbers after adsorption onto the PE fiber surface. This shift reflects the interfacial constraint effect imposed by the PE matrix on the MWCNTs, indicating significant physical interactions between them. Under this interaction, the intensities of the -CH- stretching vibration peaks at 2848 cm^−1^ and 2879 cm^−1^ in PE fibers also change markedly. These spectroscopic features collectively confirm that MWCNTs are tightly coated onto the PE fiber surface through physical interactions, forming an effective interfacial bonding between the fibers and the nanocarbon materials [32].

## 4. Discussion

**Figure 17:**
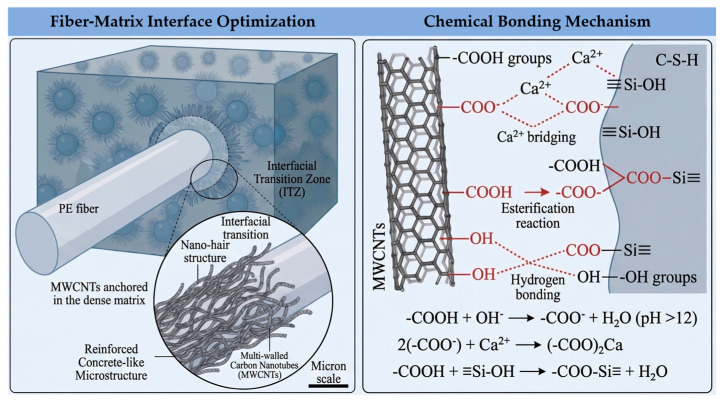
Mechanism diagram of MWCNTs enhancing ECC.

MWCNTs form nanoscale villous structures on the surface of PE fibers, which are deeply embedded in the cementitious matrix, thereby creating a micro-anchoring mechanism analogous to that of reinforced concrete [33]. The oxygen-containing functional groups on the surface of MWCNTs, including carboxyl (-COOH), hydroxyl (-OH), and carbonyl (C=O), can interact with Ca^2+^ ions and C-S-H gel generated during cement hydration; the corresponding reaction mechanisms are illustrated in Figure 17. Under highly alkaline conditions (pH > 12) in the cement paste, -COOH groups are fully dissociated into carboxylate ions (-COO^−^) according to the reaction: -COOH + OH^−^ → -COO^−^ + H_2_O. Subsequently, these carboxylate ions form ionic bonds with Ca^2+^: 2(-COO^−^) + Ca^2+^ → (-COO)_2_Ca, resulting in a highly stable chemical linkage. In addition, the -COOH groups on the MWCNT surface can react with silanol groups (≡Si-OH) on the C-S-H gel surface: -COOH + ≡Si-OH → -COO-Si≡ + H_2_O.

In summary, MWCNTs-modified PE fibers significantly enhanced the bridging stress and slip-hardening behavior of individual fibers, thereby strengthening the interfacial bonding between the fibers and the cementitious matrix and ultimately improving the tensile strength and strain capacity of cement-based materials.

## 5. Conclusions

This study employs different types of MWCNTs as modifying agents to functionalize the surface properties of PE fibers under varying immersion durations, thereby enhancing the interfacial performance between PE fibers and the cementitious matrix. The main conclusions are as follows:

(1) MWCNTs-modified PE fibers improved both the single-fiber pull-out strength and the mechanical properties of the fiber–cementitious composites. The enhancement became more pronounced with increasing fiber immersion time, while the modification effect remained nearly unchanged after 8 h of immersion. Compared with unmodified fibers, the single-fiber pull-out strength of PE fibers modified with MWCNTs-A, MWCNTs-OH, and MWCNTs-COOH increased by 26.19%, 33.33%, and 35.71%, respectively. The tensile strength was improved by 13.62%, 15.53%, and 15.74%, and the three-point bending strength by 6.54%, 6.68%, and 6.82%, respectively, whereas the compressive strength showed no significant change.
(2) The contact angle of MWCNTs-modified PE fibers is influenced by immersion time. With increasing immersion duration, the amount and uniformity of MWCNTs deposition on the PE fiber surface increase, leading to a gradual reduction in contact angle. When the immersion time reaches 8 h, the contact angle stabilizes. MWCNTs-A, MWCNTs-OH, and MWCNTs-COOH modified PE fibers reduce the contact angle by 26.37%, 25.15%, and 27.59%, respectively, effectively decreasing the hydrophobicity of the PE fiber surface.
(3) In this study, mixed MWCNTs were successfully and uniformly coated onto the surface of PE fibers via hydrophobic interactions. Raman spectroscopy results indicate the appearance of new characteristic peaks at 1344 cm^−1^ and 1583 cm^−1^ in the M-PE fibers, confirming effective physical bonding between MWCNTs and PE fibers. EDS results show that the oxygen content on the surface of M-PE fibers increases by 242.27%, indicating the presence of abundant oxygen-containing functional groups, which improves the chemical inertness of the PE fiber surface. After modification, the surface hydrophobicity of M-PE fibers is significantly reduced, with the contact angle decreasing from 164.2° to 118.4°, corresponding to a reduction of 27.9%.

## Figures and Tables

**Table 1:** Fiber performance parameters.

Fiber	Length/mm	Diameter/μm	Density/(g/cm^3^)	Tensile Strength/MPa	Elastic Modulus/GPa
PE	18	24	0.97	3000	116

**Table 2:** Multi-walled carbon nanotubes performance parameters.

Multi-Walled Carbon Nanotubes	Inner Diameter /nm	Outer Diameter /nm	Length /μm	Specific Surface Area/(m^2^/g)	Density/(g/cm^3^)	Functional Group Content/%	Purity /%
MWCNTs-A	3–5	8–15	3–12	>233	0.15	0	>95
MWCNTs-OH	2–5	4–8	10–20	350	0.27	2	>95
MWCNTs-COOH	2–5	8–15	30–50	210–250	0.27	2	>95

**Table 3:** Mix proportions of cement-based materials (kg/m^3^).

Cement	Sand	Fly Ash	Water	Superplasticizer
1432	420	143	300	22

**Table 4:** Experimental grouping.

Group	MWCNTs	Time/h	Group	MWCNTs	Time/h
PE	/	0	M-PE-OH-8	-OH	8
M-PE-A-2	-A	2	M-PE-OH-24	-OH	24
M-PE-A-4	-A	4	M-PE-COOH-2	-COOH	2
M-PE-A-8	-A	8	M-PE-COOH-4	-COOH	4
M-PE-A-24	-A	24	M-PE-COOH-8	-COOH	8
M-PE-OH-2	-OH	2	M-PE-COOH-24	-COOH	24
M-PE-OH-4	-OH	4			

**Table 5:** Results of monofilament pull-out tests.

Group	Pull Out the Load/N	Pull-Out Strength/MPa	Group	Pull Out the Load/N	Pull-Out Strength/MPa
PE	0.42	0.93	M-PE-OH-8	0.56	1.24
M-PE-A-2	0.44	0.97	M-PE-OH-24	0.58	1.28
M-PE-A-4	0.49	1.08	M-PE-COOH-2	0.46	1.02
M-PE-A-8	0.53	1.17	M-PE-COOH-4	0.48	1.06
M-PE-A-24	0.56	1.24	M-PE-COOH-8	0.57	1.26
M-PE-OH-2	0.45	1.00	M-PE-COOH-24	0.59	1.30
M-PE-OH-4	0.47	1.04			

**Table 6:** Results of uniaxial tensile tests.

Group	7 d Strength/MPa	7 d Strain/%	28 d Strength/MPa	28 d Strain/%
PE	3.81	2.94	4.70	4.70
M-PE-A-2	4.08	3.41	5.03	5.91
M-PE-A-4	4.21	3.78	5.22	6.77
M-PE-A-8	4.36	4.09	5.34	7.02
M-PE-A-24	4.37	4.14	5.38	7.08
M-PE-OH-2	4.17	3.33	5.10	5.72
M-PE-OH-4	4.23	3.8	5.25	6.63
M-PE-OH-8	4.38	4.12	5.43	6.98
M-PE-OH-24	4.43	4.21	5.46	7.03
M-PE-COOH-2	4.12	3.48	5.06	5.97
M-PE-COOH-4	4.28	3.71	5.31	6.58
M-PE-COOH-8	4.39	4.17	5.44	7.07
M-PE-COOH-24	4.41	4.23	5.42	7.12

**Table 7:** Results of the three-point bending test.

Group	7 d Strength/MPa	7 d Deflection/mm	28 d Strength/MPa	28 d Deflection/mm
PE	22.11	1.83	28.43	2.13
M-PE-A-2	22.83	2.25	29.11	2.59
M-PE-A-4	23.47	2.52	29.71	2.79
M-PE-A-8	23.77	2.69	30.29	3.04
M-PE-A-24	23.82	2.72	30.32	3.11
M-PE-OH-2	22.91	2.28	29.21	2.64
M-PE-OH-4	23.52	2.59	29.88	2.91
M-PE-OH-8	23.88	2.77	30.33	3.17
M-PE-OH-24	23.96	2.81	30.39	3.23
M-PE-COOH-2	23.02	2.32	29.16	2.68
M-PE-COOH-4	23.48	2.56	29.82	2.85
M-PE-COOH-8	23.91	2.74	30.37	3.19
M-PE-COOH-24	23.94	2.83	30.41	3.18

**Table 8:** Contact angle test results of MWCNTs modified PE fibers.

Time	Contact Angle	M-PE-A/°	M-PE-OH/°	M-PE-COOH/°
0 h	Left contact angle	163.9	163.9	163.9
	Right contact angle	164.5	164.5	164.5
	Contact angle	164.2	164.2	164.2
2 h	Left contact angle	129.6	126.9	121.7
	Right contact angle	135.5	125.7	122.7
	Contact angle	132.6	126.3	122.2
4 h	Left contact angle	127.4	125.8	120.4
	Right contact angle	125.2	124.2	122.8
	Contact angle	126.3	125.0	121.6
8 h	Left contact angle	119.1	125.3	118.7
	Right contact angle	122.6	120.4	119.1
	Contact angle	120.9	122.9	118.9
24 h	Left contact angle	120.6	124.1	117.8
	Right contact angle	117.5	119.9	118.1
	Contact angle	119.1	122.0	118.0

**Table 9:** Test results of M-PE Fiber EDS.

Group	Element	Mass Percentage/wt%	Atomic Percentage/%
PE	C	97.09	97.80
	O	2.91	2.20
M-PE-A	C	97.11	97.81
	O	2.89	2.19
M-PE-OH	C	95.48	96.57
	O	4.52	3.43
M-PE-COOH	C	85.72	88.88
	O	14.28	11.12
M-PE	C	90.04	92.33
	O	9.96	7.67

## Data Availability

Dataset available on request from the authors.
